# miRNet 2.0: network-based visual analytics for miRNA functional analysis and systems biology

**DOI:** 10.1093/nar/gkaa467

**Published:** 2020-06-02

**Authors:** Le Chang, Guangyan Zhou, Othman Soufan, Jianguo Xia

**Affiliations:** Department of Human Genetics, McGill University, Montreal, Quebec, Canada; Institute of Parasitology, McGill University, Montreal, Quebec, Canada; Institute of Parasitology, McGill University, Montreal, Quebec, Canada; Department of Human Genetics, McGill University, Montreal, Quebec, Canada; Institute of Parasitology, McGill University, Montreal, Quebec, Canada; Department of Animal Science, McGill University, Montreal, Quebec, Canada

## Abstract

miRNet is an easy-to-use, web-based platform designed to help elucidate microRNA (miRNA) functions by integrating users' data with existing knowledge via network-based visual analytics. Since its first release in 2016, miRNet has been accessed by >20 000 researchers worldwide, with ∼100 users on a daily basis. While version 1.0 was focused primarily on miRNA-target gene interactions, it has become clear that in order to obtain a global view of miRNA functions, it is necessary to bring other important players into the context during analysis. Driven by this concept, in miRNet version 2.0, we have (i) added support for transcription factors (TFs) and single nucleotide polymorphisms (SNPs) that affect miRNAs, miRNA-binding sites or target genes, whilst also greatly increased (>5-fold) the underlying knowledgebases of miRNAs, ncRNAs and disease associations; (ii) implemented new functions to allow creation and visual exploration of multipartite networks, with enhanced support for *in situ* functional analysis and (iii) revamped the web interface, optimized the workflow, and introduced microservices and web application programming interface (API) to sustain high-performance, real-time data analysis. The underlying R package is also released in tandem with version 2.0 to allow more flexible data analysis for R programmers. The miRNet 2.0 website is freely available at https://www.mirnet.ca.

## INTRODUCTION

Gene expression in eukaryotes is a complex process controlled by many factors functioning at epigenetic, transcriptional or post-transcriptional levels (1,2). Over the past two decades, the broad applications of comprehensive molecular profiling technologies have enabled us to study gene expression in various biological processes and disease conditions. However, our understanding of the underlying regulatory mechanisms remains incomplete. It has become clear that in order to address this issue, it is critical to adopt a systems biology approach to integrate all important players (3–5). Network-based approaches have received wide attention as they can abstract and integrate different types of information in a format that is often intuitive and interpretable (6–8). Based on this concept, we developed miRNet version 1.0 to help illustrate the ‘multiple-to-multiple’ relationships (i.e. one miRNA can regulate multiple genes and one gene can be regulated by multiple miRNAs) through network-based visualization of miRNA-target gene interactions coupled with improved functional analysis (9,10). However, the interplays between miRNAs and target genes represent only the starting points toward understanding the roles that miRNAs play at cellular level. In particular, miRNAs can regulate gene regulatory networks through feedback or feedforward loops (11), for instance, by adjusting expression of transcription factors (TFs) which in turn exert effects on their corresponding target genes. Such higher-order interactions are not captured in miRNet version 1.0.

The past few years have witnessed several trends in miRNA research. A growing number of studies have employed systems biology approaches either experimentally by employing multi-omics measurements or computationally by including other key factors such as miRNA–lncRNA–gene (12), miRNA–TF–gene (4) or miRNA–gene–disease (13) to better understand miRNA regulatory mechanisms. Another growing area of research is precision medicine, in which the characteristic gene expression patterns of a particular patient can be interpreted by his or her own genetic mutations to inform treatment or prevention plan (14). For instance, SNPs in miRNA and miRNA-binding sites have been found to be associated with several diseases (15). The complex interplays amongst different functional elements can be captured using multipartite networks to reveal a more holistic picture. However, integrating multiple data types and interpreting these results at a systems level is challenging (16). Building such networks requires manual curation of data from multiple databases and powerful network visualization support to aid researchers in navigation and understanding.

Since the release of miRNet version 1.0, many new features and components have been gradually introduced based on users’ feedback and developments in the field. For instance, tissue and cellular contexts are important for interpretation of miRNA-gene interactions. To support this need, we have implemented tissue-specific filters based on their expression profiles (17). In addition, current bioinformatics tools focus primarily on human and other model organisms. To facilitate miRNA research in species important for agriculture and veterinary medicine, we have added support for cows, pigs and chickens following well-established protocols (18). For researchers interested in exploring potential regulatory roles of miRNAs derived from pathogens such as parasitic helminths (19), viruses (20) or other sources, we have added support for reported or putative xeno-miRNAs (21). A continuous effort has been to keep current with new releases of its underlying databases as well as to maintain backward compatibility. This effort has triggered several rounds of code refactoring to achieve a more robust and modular design, with computational intensive tasks distributed among different servers through microservices (22). The latter technique also helps address computational bottlenecks with bigger databases and growing user traffic. The user-friendly web interface is mainly used by clinicians and bench biologists with little to no programming skills. While for bioinformaticians or tool developers, it may be more meaningful to directly access miRNet's functionality through its underlying R code or a well-defined application programming interface (API).

To address these emerging bioinformatics needs and challenges, we developed miRNet version 2.0 to allow users to easily create complex miRNA-centric networks for systems-level interpretation of miRNA functions and gene regulations. The 2.0 release captures all the aforementioned updates since 2016 and represents a solid step toward network-based data integration for miRNA systems biology. A more detailed description of each of these updates and changes in miRNet 2.0 is given below.

## PROGRAM DESCRIPTION AND METHODS

### Overview of miRNet 2.0 framework

**Figure 1.**
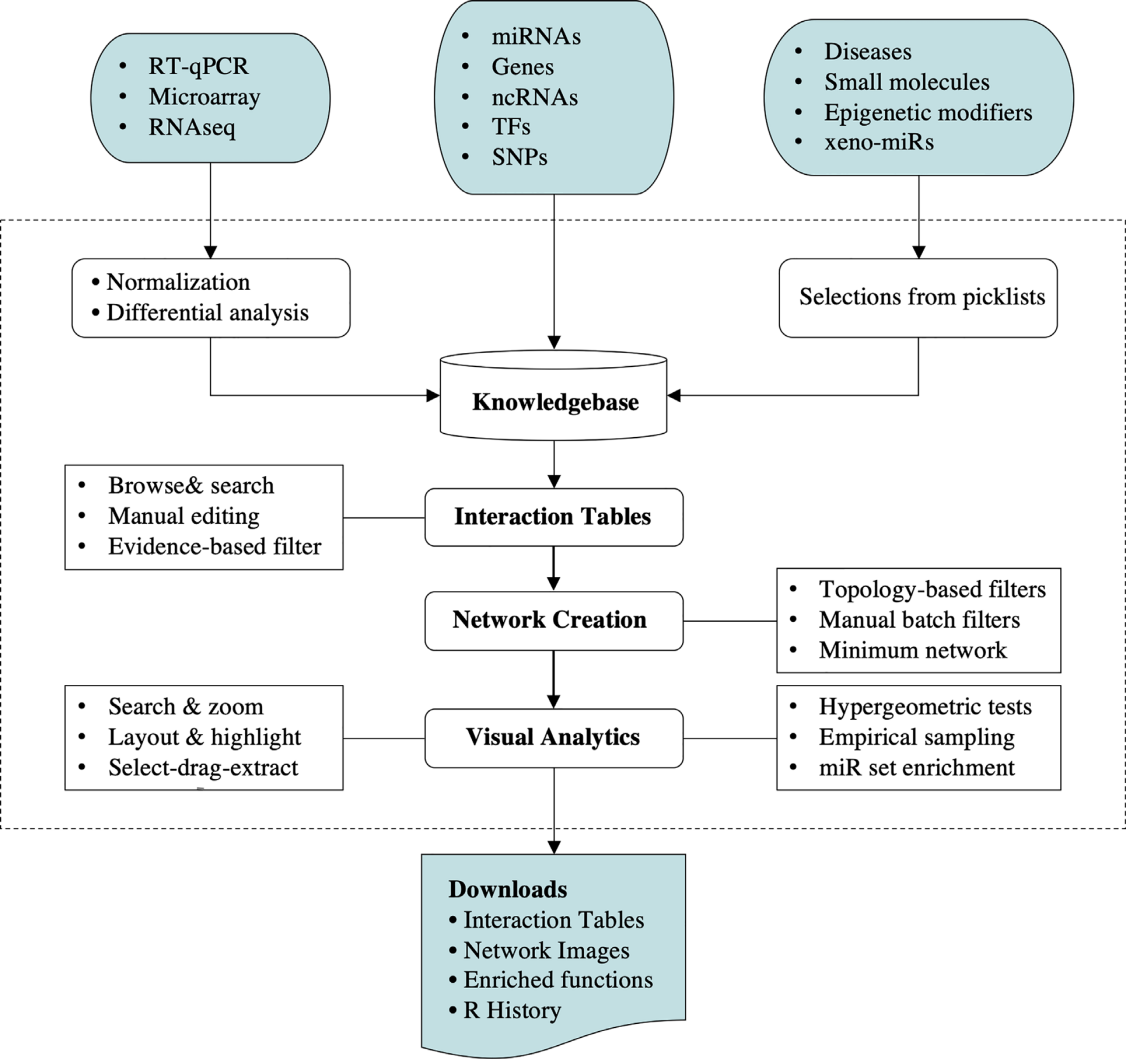
Overview of miRNet 2.0 workflow. Users can upload different data types or select queries from built-in databases to start analysis. The input will be mapped to the underlying knowledgebases to create interaction tables and networks. The visualization page allows users to intuitively explore the networks using different layout algorithms as well as to perform topology or functional analysis.

The main workflow of miRNet 2.0 is summarized in Figure 1. There are three main steps—data input, network creation and network visual analytics. To maintain a flexible and modular design, we have organized the main functions into 12 modules based on input types. The ‘miRNAs’ module allows users to connect miRNAs with target genes, TFs, ncRNAs *etc*.; the ‘Genes’ and ‘TFs’ modules link the corresponding inputs to their partners within the context of known interactions among miRNAs, genes and TFs; the ‘SNPs’ module maps SNPs to the above key players themselves or their binding sites. The remaining modules follow a similar procedure by mapping users’ inputs to their corresponding miRNA associated interaction partners. To start, users must click a circular button from the miRNet homepage to enter the corresponding data upload page. Two general data formats are accepted: a list of miRNAs, SNPs, genes, small molecules *etc*., or an expression table generated from qPCR, microarray or RNAseq experiments. In the latter case, well-established differential expression analysis will be applied to identify significant miRNAs or genes as new input lists. In the second step, the input lists will be mapped to the underlying knowledgebases to create one or more interaction tables and networks. Many functions are available to allow users to further customize or refine the networks. In the third step, the results are presented as interactive networks for visual exploration. Users can easily search, zoom, highlight or perform functional enrichment analysis on selected regions of interest. In the following sections, we will focus primarily on the new and improved features introduced in version 2.0. Other features can be found in our prior publications (9,10,21).

### Knowledgebase update and creation

#### Knowledgebase for network creation

We have put considerable efforts into keeping miRNet's underlying knowledgebases up to date. miRNet 2.0 can automatically recognize different versions of miRBase IDs, as well as link pre-miRNAs to their mature forms based on the miRBaseConverter R package (23). We have updated the miRNA interaction knowledgebase based on the latest releases from major miRNA annotation databases including miRBase (24), miRTarBase (25), TarBase (26), HMDD (27) *etc*. The human tissue-specific miRNA annotations are based on TSmiR (28) and IMOTA (17) databases, and the human exosomal miRNA annotations are from ExoCarta (29). The interactions among miRNAs, TFs and genes are obtained from TransmiR 2.0 (30), ENCODE (31), JASPAR (32) and ChEA (33). For miR-SNPs, we have used ADmiRE (34), PolymiRTS (35) and SNP2TFBS (36) to obtain SNP information in miRNA genes, miRNA-binding sites and TF-binding sites. We have also systematically collected the reported xeno-miRNAs together with their putative targeted genes into xeno-miRNet (21), which is now integrated in miRNet 2.0. Finally, we have expanded the miRNA-lncRNA interactions to include all other major ncRNAs including circRNA, ceRNA, pseudogene and sncRNA based on starBase (37). These data can be downloaded from the miRNet ‘Resources’ page as plain text files.

#### Knowledgebase for network interpretation

For network analysis, it is important to be able to interpret the interactions in addition to their visualization. Enrichment analysis plays a significant role in this respect. Applying conventional enrichment analyses such as hypergeometric tests on target genes are known to be biased (38,39). In miRNet 1.0, we implemented an algorithm based on empirical sampling for enrichment analysis using GO, KEGG or Reactome pathways (38). Another effective approach is to perform enrichment analysis directly at miRNA levels (39). To support this type of analysis, we have added six miRNA-set libraries including miRNA–function, miRNA–disease, miRNA–TF, miRNA–cluster, miRNA–family and miRNA–tissue based on TAM 2.0 (40). In summary, miRNet 2.0 provides four query types (all genes, highlighted genes, all miRNAs, highlighted miRNAs), two enrichment algorithms (hypergeometric tests and empirical sampling), nine annotation libraries (three gene-set libraries and six miRNA-set libraries), representing the most comprehensive support to understand collective functions of miRNAs. Their potential applications are showcased in recent studies to compare miRNA changes specific to different tissues in pancreatic ductal adenocarcinoma (41) and to identify enriched miRNA families in a study comparing genetic variants between Alzheimer's disease and cancers (42).

### Enabling flexible user input

Significant efforts have been made to provide an intuitive interface that permits the integration of miRNAs into different types of interaction networks. From the homepage, users can enter their queries by: (a) uploading a list of miRNAs, ncRNAs, genes, TFs or SNPs; (b) selecting a list from our built-in databases such as diseases, small compounds, epigenetic modifiers *etc*. (c) uploading a miRNA or gene expression table generated from RT-qPCR, microarray or RNAseq or (d) uploading multiple queries of different input types. Here, we will introduce new features for several common scenarios.

#### From miRNAs to networks

In miRNet 1.0, miRNA–targets mapping was limited to target genes based on experimentally validated interaction information. However, increasing evidence has shown that miRNAs participate in complex networks through interactions with other functional elements to exert effects on cell biology and human diseases (12). For instance, lncRNAs can act as miRNA ‘sponge’ and compete with target mRNAs, thus increasing the expression level of mRNAs (43). In version 2.0, users can select one or multiple targets from the ‘Targets’ dropdown list and miRNet will automatically map miRNAs to those selected targets. Users can further include protein-protein interactions (PPI) in the target networks based on several well-established PPI databases (44–46).

#### From TFs to networks

miRNAs and TFs can cooperate to tune gene expression, or mutually regulate each other in feedback loops (4,47). Consequently, we have added a new module to allow users to include TFs into analysis. Users can simply upload their TF list, miRNet will automatically map the TFs to all potential targets (miRNAs and/or genes) and return as TF–miRNA and/or TF–gene interaction tables. The interactions will then be further integrated into networks for visual exploration. With the updated miRNA module and the addition of the TF module, miRNet 2.0 allows users to easily create miRNA-TF coregulatory networks from either a list of miRNAs or a list of TFs of interest.

#### From SNPs to networks

Mutations in mature miRNAs or their binding sites could significantly change their targeting abilities and dysregulate the expression of many genes simultaneously, whereas variations in primary or precursor miRNAs could alter the expression levels of mature miRNAs by affecting miRNA processing (48,49). In miRNet 2.0, we have added a new module to support the analysis of SNPs within the context of miRNA-target gene interactions. Users can upload a list of SNPs from the SNPs upload page. miRNet currently accepts either rsIDs or genomic coordinates based on the human reference genome build GRCh37. The uploaded lists are then mapped to miRNAs and/or their target genes. Following this step, users can visually explore their data in the network visualization page.

#### Uploading multiple queries

The Multiple Query Types module complements miRNet's single type analysis modules by permitting the identification of novel connections amongst multiple types of user input. The module currently supports ten input types shown in a dialog when users click the central circular button at the home page. After selecting the input types of interest, users simply copy-and-paste their query lists (miRNAs, genes, TFs, lncRNAs, pseudogenes, circRNAs, sncRNAs) or select from picklists (diseases, small compounds and epigenetic modifiers). The uploaded lists are then mapped to the internal knowledgebases and proceed with the workflow as described in other modules.

### Enhancing network visual analytics

#### Network creation and customization

The default networks are created by searching for direct interaction partners in the interaction knowledgebases. These are generally known as first-order interaction networks. When there is a large number of queries (seeds), it is reasonable to focus only on the interactions among those seeds (i.e. zero-order networks). However, many seeds could become orphan nodes when switching directly to zero-order networks. A ‘gentle’ approach is to extract, from the first-order network, a minimal subnetwork that maximally connects those seeds. In miRNet 2.0, we have added the support for computing minimum subnetworks based on the prize-collecting Steiner Forest (PCSF) algorithm (50), as well as several other empirical refining methods (available under ‘Network Tools’) based on shortest paths, batch filtering, node degree or betweenness values. The results can be downloaded as pair-wise interaction tables or graph files.

#### Network visualization and layout

**Figure 2.**
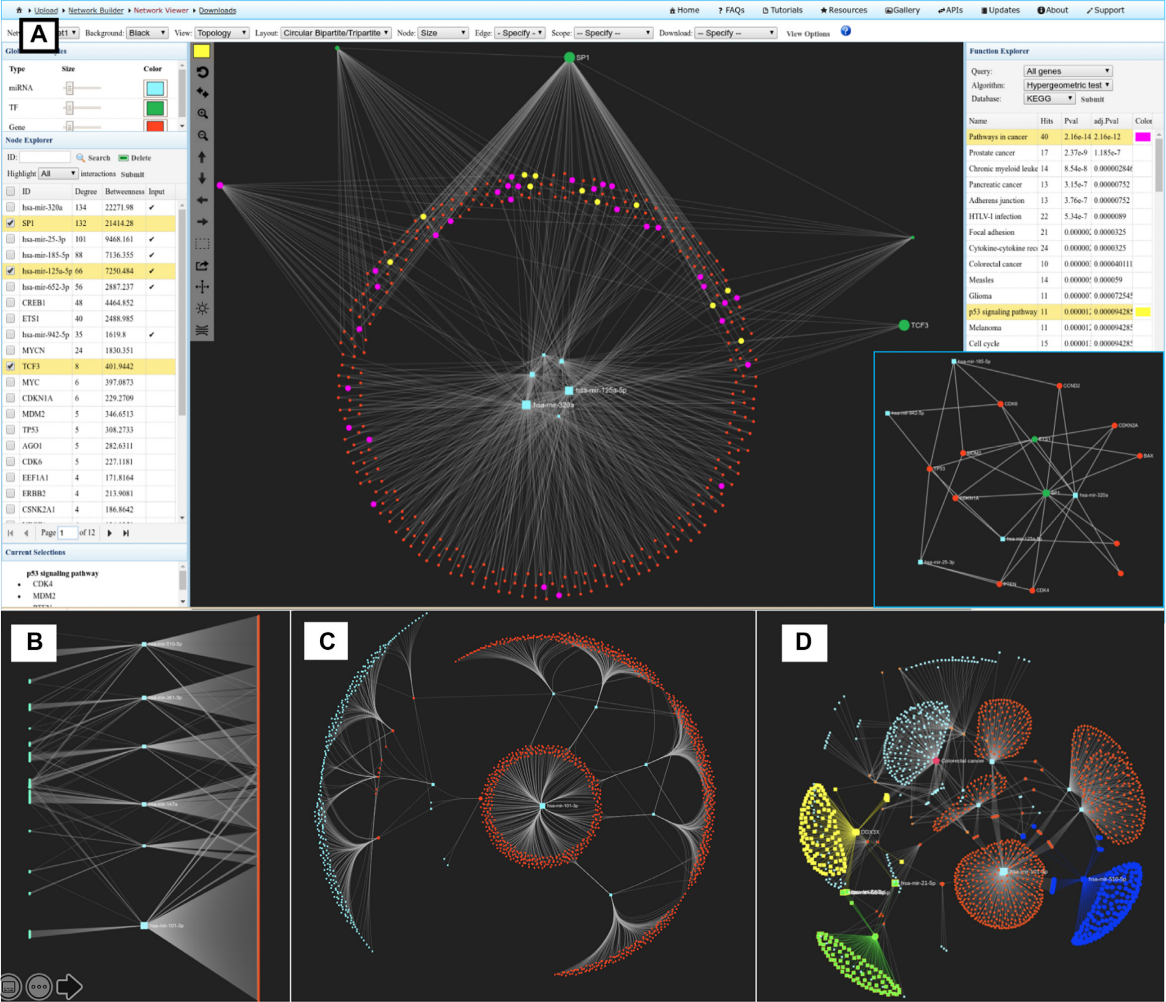
Screenshots of the Network Visualization page showing the main features and several network layouts. (**A**) A typical view of the page. The central panel shows a network in Circular-tripartite layout, and the surrounding panels provide functions for network analysis and customization. For instance, users can perform enrichment analysis or module analysis on this network. An extracted network module was displayed at bottom right. (**B**) Linear-tripartite layout. (**C**) Concentric layout with edge bundling. (**D**) Backbone layout with several modules highlighted in different colors. More details of each layout are described in the main text.

miRNet 2.0 provides a wide array of options to help improve visual exploration of miRNA-centric interaction networks. During the network creation stage, users can refine the network by applying different filters on interaction tables or networks. At the network visualization page, users can specify node styles based on their types, reduce node overlap, or perform edge bundling *etc*. The resulting network can be further improved using different layout algorithms. Over ten network layout algorithms have been implemented, including Force-Atlas, Fruchterman-Reingold, Circular, Graphopt, Large Graph, Random, Circular Bipartite/Tripartite, Linear Bipartite/Tripartite, Concentric and Backbone. The latter four algorithms are designed for complex networks consisting of multiple node types (miRNAs, genes, TFs *etc*.). The bipartite/tripartite layout provides a straightforward abstraction of the relationships between different types of molecular entities by emphasizing the data type of each node (51). When there are multiple node types, we recommend visualizing the network in either circular bipartite/tripartite (Figure 2A) or linear bipartite/tripartite layout (Figure 2B) followed by applying the ‘reduce node overlap’ algorithm. To enable better understanding of a particular key node, we have added the Concentric layout (52). This layout arranges nodes in concentric circles around a node of interest (i.e. the focal node) in the middle (Figure 2C). The order of the circles represents the degree level of their interactions. By arranging nodes in this fashion, it enables a better understanding of how the focal node relates to the rest of the graph. By default, the focal node is the node with the highest degree value. Users can manually specify the key node by selecting it in the Node Explorer table or by double clicking on it in the network. Another new addition is the Backbone layout which is very effective in revealing hidden patterns in medium and large networks. The algorithm calculates layout after applying sparsification on the network by only including the most embedded edges (53). This process helps uncover hidden modules based on edge density by putting more emphasis on the structure of graph layout (Figure 2D).

### Improving transparency/reproducibility and web APIs

Except for the interactive visualization step, which is executed on users’ browsers, all other data analysis steps including mapping, filtering, network creation and customization are performed by the corresponding R functions on our cloud server. To enable more transparent data analysis, we have released the underlying R package (https://github.com/xia-lab/miRNetR), and added a ‘Download’ page in the web application to allow users to download the R command history and results tables generated during their analysis sessions. The R history contains all function calls with user-selected parameters. We hope that the R package together with the R command history will allow users to track each step of their analysis in a form (R script) that can be easily shared and reproduced, complementing the web-based platform. We have also implemented RESTful APIs to allow tool developers to submit their query lists programmatically as external requests. While offering open access to miRNet 2.0 resources, APIs give a level of abstraction and hide complexity from programmers. The currently available APIs are shown in Table 1. More APIs will be added based on users’ feedback.

**Table 1.** List of APIs and programmatic access endpoints on the miRNet server. The API base for miRNet 2.0 is http://api.mirnet.ca, which can be visited to view a detailed documentation

Endpoint	HTTP method	Input	Description
**base/table/mir**	POST	Organism, miRNA ID type, target type, miRNA list	Get experimentally validated table results of the miRNA-target interactions (forward mapping)
**base/table/gene**	POST	Organism, gene ID type, gene list	Get experimentally validated table results of the miRNA-gene (mRNA, TF, lncRNA) interactions (reverse mapping)
**base/function/mir**	POST	Organism, miRNA ID type, target type, miRNA list, algorithm, database	Get functional enrichment results
**base/function/gene**	POST	Organism, gene ID type, gene list, algorithm, database	Get functional enrichment results
**base/graph/mir**	POST	Organism, miRNA ID type, target type, miRNA list	Get graph of miRNA–target interactions (json format)
**base/graph/gene**	POST	Organism, gene ID type, gene list	Get graph of miRNA–target interactions (json format)

## USE CASES

To further demonstrate the utility of these new features in miRNet 2.0, we used data from a multiple sclerosis (MS) study aiming to identify the role of miRNA and TF co-regulatory networks in the pathogenesis of MS (54). In this study, the miRNA target analysis and TF target analysis were performed by searching several miRNA–gene, TF–gene and TF–miRNA databases. The network was manually built by using Cytoscape (55). We reconstructed and visualized the network in miRNet 2.0 by using the miRNA module. The resulting network is comprised of 2414 nodes (TF: 5; Gene: 2403; miRNA: 6) and 2798 edges. For better visual exploration, a degree cutoff 1.0 was applied. As shown in Figure 2A, the TF-miRNA co-regulatory networks is displayed at the center of the Network Viewer page in Circular Tripartite layout. It illustrates various interactions between miRNAs (inner zone), genes (middle layer) as well as TFs (outer layer). The nodes are sorted by degree centrality measures in the *Node Explorer* table. In this case, miRNet 2.0 confirmed the detection of important nodes according to their degree measures. Among the top nodes, hsa-miR-125a-5p (degree = 66) has been frequently associated with MS, while *SP1* (degree = 132) and *TCF3* (degree = 8) have been reported in the transcriptional regulations of MS (54). We also performed functional enrichment and module analysis on the whole network. The results of functional enrichment analysis using KEGG database are displayed in the *Function Explorer* table. For instance, cytokine-cytokine receptor interaction pathway (adj. *P*-value = 3.25 × 10^−5^) and p53 signaling pathway (*adj*. *P*-value = 9.43 × 10^−5^) were significantly enriched, which were not reported by the original study but the results have been supported by other publications (56). Figure 2A (lower right corner) shows an example of an extracted module (p53 signaling pathway) after manual increase of the edge thickness. Compared to the original network, this module is much more digestible while keeping the important nodes and connections (e.g. hsa-miR-125a-5p and *SP1*). This use case highlights that with only a few mouse clicks, users can easily create comprehensive regulatory networks to gain a more holistic view of miRNA-mediated regulations as well as to extract important modules for more in-depth analysis.

### Comparison with miRNet 1.0 and other web-based tools

Several excellent web-based tools have been developed for miRNA network analysis, including miRTargetLink (57), MIENTURNET (58), Arena-Idb (59), and starBase (37). Detailed comparison between these tools and miRNet 2.0, as well as its previous version is shown in Table 2. Particularly, miRTargetLink, MIENTURNET and Arena-Idb can assist researchers in understanding miRNAs and their targets through a network-based visualization method based on predicted or experimentally validated miRNA–target interactions; while starBase is the most comprehensive miRNA–mRNA and miRNA–ncRNA interaction database based on CLIP-Seq experiments. In comparison, miRNet 2.0 is a high-performance, easy-to-use web application which offers the most comprehensive support for real-time, interactive miRNA network analytics in ways that no other tools currently can. More than 15 databases and over 10 graph layout algorithms have been integrated to facilitate knowledge discovery and hypothesis generation. The companion R package and APIs have been developed to permit transparent and reproducible analysis as well as to reach a broader user base. In summary, miRNet 2.0 caters for both bench researchers as well as bioinformaticians by providing an interactive and integrative platform for miRNA-centric systems biology.

**Table 2.** Comparison of the main features of miRNet (versions 1.0–2.0) with other web-based or web-enabled tools. Symbols used for feature evaluations with ‘√’ for present, ‘−’ for absent, and ‘+’ for a more quantitative assessment (more ‘+’ indicate better support)

	miRNet					
Tool name	2.0	1.0	miRTargetLink	MIENTURNET	Arena-Idb	starBase
**Data input and processing**						
**Species #**	11	8	1	6	1	23
**Target genes**						
Experimental	+++	++	++	+	++	+++
Predicted	√	√	√	√	√	√
**Other targets & associations**						
miR-SNP	√	−	−	−	−	−
TF	√	−	−	−	−	−
ncRNA	+++	+	−	−	+	++++
xeno-miRNA	√	−	−	−	−	−
Disease	+++	++	−	−	++	++
Epigenetic modifier	√	√	−	−	−	−
Small molecule	√	√	−	−	−	−
Expression profiling	√	√	−	−	−	−
**Enrichment analysis**						
Hypergeometric tests	√	√	√	√	−	√
Empirical sampling	√	√	−	−	−	−
miR-set enrichment	√	−	−	−	−	−
**Network visual analytics**						
Multiple query types	√	−	−	−	−	−
Integration with PPI network	√	−	−	−	−	−
Multipartite network visualization	√	−	−	−	−	−
Subnetwork extraction	√	−	−	−	−	−

**URL links:**

miRTargetLink: https://ccb-web.cs.uni-saarland.de/mirtargetlink/

MIENTURNET: http://userver.bio.uniroma1.it/apps/mienturnet/

Arena-Idb: http://ncrnadb.scienze.univr.it/sites/arenaidb/

starBase: http://starbase.sysu.edu.cn/index.php

## CONCLUSIONS

Over the past few years, miRNA research has gradually evolved from target identification toward understanding the regulatory mechanisms underlying their systems level effects. However, very few user-friendly bioinformatics tools are available to support this objective. To address this gap, we have developed miRNet version 2.0 to assist researchers to easily create miRNA-centric multiplex networks integrating key players involved in gene regulation as well as other molecules of interest. During this process, we have greatly expanded the underlying knowledgebases and added new libraries on TFs, SNPs, ncRNAs and PPIs to provide a rich context for analysis, hypothesis generation and mechanistic insights. We have also implemented new graph mining functions and layout algorithms tailored to complex multipartite network creation, customization and visualization. To sustain real-time intuitive data analysis, we have completely revamped the web interface, optimized the workflow, introduced APIs and microservices to enable high-performance computing and visualization. A limitation of miRNet is its static and qualitative nature of the current network analysis. It is important to keep in mind that miRNA functions are highly dependent on the context (abundance, location, cell type, cell state e*tc*.) and the effects can be dynamic and transient to confer robustness to biological processes (60). We believe that miRNet 2.0 will continue to be an invaluable bioinformatics asset for researchers in miRNA systems biology.

## DATA AVAILABILITY

The miRNet 2.0 web server can be freely accessed at https://www.mirnet.ca. The web APIs can be accessed from http://api.mirnet.ca. The miRNetR is available on Github (https://github.com/xia-lab/miRNetR).
